# Afatinib alone and in combination with vinorelbine or paclitaxel, in patients with HER2-positive breast cancer who failed or progressed on prior trastuzumab and/or lapatinib (LUX-Breast 2): an open-label, multicenter, phase II trial

**DOI:** 10.1007/s10549-021-06449-4

**Published:** 2022-02-09

**Authors:** Tamas Hickish, Ajay Mehta, Mei-Ching Liu, Chiun-Sheng Huang, Rajendra Singh Arora, Yuan-Ching Chang, Youngsen Yang, Vladimir Vladimirov, Minish Jain, Janice Tsang, Karine Pemberton, Behbood Sadrolhefazi, Xidong Jin, Ling-Ming Tseng

**Affiliations:** 1 Royal Bournemouth Hospital/Bournemouth University, Castle Ln E, Bournemouth, BH7 7DW UK; 2 Central India Cancer Research Institute, Nagpur, India; 3 Koo Foundation Sun Yat-Sen Cancer Center, Taipei, Taiwan; 4 National Taiwan University Hospital, Taipei, Taiwan; 5 Sujan Cancer & Surgical Hospital, Amravati, India; 6 Mackay Memorial Hospital, Taipei, Taiwan; 7 Division of Hematology-Oncology, Department of Internal Medicine, Taichung Veterans General Hospital, Taichung, Taiwan; 8 Internal Medicine, College of Medicine, China Medical University, Taichung, Taiwan; 9 Pyatigorsk Oncology Dispensary, Pyatigorsk, Russia; 10 Ruby Hall Clinic, Pune, Maharashtra India; 11 Li Ka Shing Faculty of Medicine, The University of Hong Kong, Pok Fu Lam, Hong Kong; 12 Boehringer Ingelheim Ltd, Bracknell, UK; 13 Boehringer Ingelheim Canada Ltd, Burlington, ON Canada; 14 Boehringer Ingelheim Pharmaceuticals Inc, Ridgefield, CT USA; 15 Taipei Veterans General Hospital, Taipei, Taiwan

**Keywords:** Afatinib, ErbB, HER2, Metastatic breast cancer, Resistance

## Abstract

**Purpose:**

Resistance to HER2 (ErbB2)-targeted therapy may be mediated by other members of the ErbB family. We investigated the efficacy and safety of the irreversible ErbB family blocker, afatinib, alone as first-line therapy in the advanced setting and in combination with vinorelbine or paclitaxel for those who progressed on afatinib monotherapy, in female patients with metastatic breast cancer who had failed or progressed on prior HER2-targeted therapy in the early disease setting.

**Methods:**

In this phase II, single-arm, two-part study (ClinicalTrials.gov: NCT01271725), patients in part A received afatinib 40 mg/day in 21-day cycles until disease progression or intolerable adverse events (AEs). Patients with progressive disease could then receive afatinib plus weekly vinorelbine 25 mg/m^2^ or paclitaxel 80 mg/m^2^ until disease progression or intolerable AEs (part B). The primary endpoint was confirmed objective response rate (RECIST v1.1).

**Results:**

Eighty-seven patients were enrolled and 74 were treated in part A (median age: 51 years [range 27–76]; 31 [42%] estrogen receptor-positive, 26 [35%] progesterone receptor-positive). Of these, 39 (53%) patients went on to receive afatinib plus vinorelbine (13 patients) or paclitaxel (26 patients) in part B. Thirteen (18%) and 12 (31%) patients achieved an objective response in parts A and B, respectively. The most common treatment-related AEs with afatinib monotherapy (any/grade ≥ 3) were diarrhea (68%/8%) and rash (49%/4%). Combination therapy was generally well tolerated, with no additive toxicity observed.

**Conclusion:**

Afatinib treatment, alone or in combination with vinorelbine or paclitaxel, was associated with objective responses in ≥ 18% of patients with metastatic breast cancer for whom prior HER2-targeted therapy has failed. Treatment-related AEs were generally manageable, with few grade ≥ 3 AEs reported.

**Trial registration:**

ClinicalTrials.gov, NCT01271725, registered 1 July 2011.

## Introduction

The human epidermal growth factor receptor 2 (HER2/Neu/ErbB2) is amplified and/or overexpressed in approximately 15% of human breast cancers [1] and plays a direct role in tumor development [2, 3]. In patients with breast cancer, HER2 amplification/overexpression is a significant predictor of shorter overall survival and time to relapse [4, 5]. Targeted [6–8] treatment against HER2 with the monoclonal antibodies trastuzumab and pertuzumab, the small molecule tyrosine kinase inhibitors lapatinib, neratinib, and tucatinib, and the antibody-chemotherapy conjugates trastuzumab emtansine and trastuzumab deruxtecan has markedly improved outcomes in patients with HER2-positive breast cancer [9–13]. These treatments are now approved and recommended for use in this setting [14, 15].

Despite the availability of anti-HER2 therapies, primary and acquired resistance to these treatments frequently occur and represent a significant clinical challenge [16–18]. Novel agents to treat patients with HER2-positive breast cancer who have exhausted all current options are therefore urgently needed. Diverse mechanisms of resistance to anti-HER2 therapy, mediated by aberrations in numerous alternative pathways, have been identified, including those involving other receptors in the ErbB family, such as the epidermal growth factor receptor ([EGFR]/ErbB1), HER3 (ErbB3), and HER4 (ErbB4) [16, 17, 19, 20]. Some evidence suggests that targeting multiple members of the ErbB family improves outcomes compared with targeting one receptor alone [12, 21], although not all studies support these findings [22].

Afatinib, an irreversible ErbB family blocker, is a specific, potent inhibitor of HER2, EGFR, and HER4, which blocks signaling from all homo- and heterodimers formed by ErbB family members [23]. We hypothesized that broad inhibition of multiple ErbB family members may help to overcome resistance to prior HER2-targeted therapy and findings from previous phase I and II trials of afatinib in HER2-positive metastatic breast cancer were encouraging [24–27]. In one phase II study, four of 35 evaluable patients (11%) experienced a partial response to afatinib monotherapy after progression on trastuzumab and 15 patients (43%) had stable disease [25]. In the neoadjuvant setting, single-agent afatinib demonstrated clinical activity comparable to that of trastuzumab and lapatinib in HER2-positive patients with locally advanced breast cancer [27].

The present trial aimed to investigate the use of afatinib, both alone and in combination with chemotherapy, in a larger population of patients with HER2-positive metastatic breast cancer who had failed previous HER2-targeted therapy. Previous studies of afatinib and vinorelbine in patients with breast cancer suggested that these two agents could be safely combined, with potential clinical benefit [24, 26]. In keeping with current use of trastuzumab [28], afatinib was to be continued beyond the point of disease progression.

## Material and methods

### Patients and study design

In this open-label, phase II, single-arm, two-part study, patients were enrolled at 27 sites across Asia and Europe (Russia, the United Kingdom, Poland, Hong Kong, Taiwan, and India). At two sites, including the site in Poland, patients were enrolled but not treated. In part A, patients received afatinib monotherapy; in part B, patients with disease progression on monotherapy during part A were eligible to receive combination therapy with afatinib and either vinorelbine or paclitaxel.

The study enrolled female patients aged 18 years or over with histologically confirmed HER2-overexpressing metastatic breast cancer. Patients must have failed or progressed on prior trastuzumab or lapatinib or trastuzumab and lapatinib combination therapy, in the neoadjuvant and/or adjuvant setting. Other inclusion criteria included at least one measurable lesion according to the Response Evaluation Criteria in Solid Tumors version 1.1 (RECIST v1.1) [29], an Eastern Cooperative Oncology Group (ECOG) performance status of 0–2, life expectancy of at least 6 months, and an archived tissue sample available for central re-assessment of HER2 status. Patients eligible for part B must also have progressed on afatinib monotherapy in the first-line metastatic setting (part A) and be eligible for treatment with vinorelbine or paclitaxel.

Key exclusion criteria were as follows: presence of active brain metastases, prior first-line therapy for metastatic breast cancer; radiotherapy (except short-course palliative radiotherapy to non-target lesions), chemotherapy, immunotherapy, any investigational drug, trastuzumab or lapatinib treatment, or surgery (other than biopsy) within 4 weeks prior to trial treatment; hormone therapy for breast cancer within 2 weeks prior to trial treatment; or prior EGFR/HER2-targeted treatment other than trastuzumab or lapatinib in the neoadjuvant and/or adjuvant setting (parts A and B) or afatinib in the first-line setting (part B).

All patients provided written informed consent. The trial was carried out in accordance with the Declaration of Helsinki and the protocol was approved by the independent ethics committees and/or institutional review boards of the participating centers. The study is registered with ClinicalTrials.gov (NCT01271725).

### Study treatment

In part A, afatinib was administered orally at a dose of 40 mg/day until disease progression or intolerable adverse events (AEs). Once disease progression occurred, patients received weekly intravenous infusions of either vinorelbine 25 mg/m^2^ or paclitaxel 80 mg/m^2^ in addition to daily afatinib until disease progression or intolerable AEs. The choice of chemotherapeutic agent was made by the investigator, providing patients were eligible for either treatment. Treatment cycles were 21 days.

For the first instance of disease progression in part A patients could receive short-course palliative radiotherapy if necessary, while continuing afatinib monotherapy. Patients could enter part B 3ؘ–4 weeks after completion of radiotherapy.

In the event of any grade ≥ 3 treatment-related AE, grade 2 diarrhea persisting for ≥ 2 consecutive days despite adequate anti-diarrheal medication/hydration, grade ≥ 2 nausea and/or vomiting persisting for ≥ 7 consecutive days despite antiemetic treatment/hydration, or grade ≥ 2 worsening of renal function, afatinib treatment was paused until the AE had recovered to grade ≤ 1 or baseline. Treatment was then resumed at a 10-mg lower dose, with a minimum dose of 20 mg (treatment was discontinued if the patient was already receiving a dose of 20 mg).

### Endpoints

The primary endpoint was confirmed objective response, defined as complete response (CR) plus partial response (PR). Secondary endpoints were as follows: best overall response (objective response with and without confirmation) during each treatment period; duration of objective response (without confirmation), defined as the time from first objective response to time of progression, death, or censoring; progression-free survival (PFS), defined for three time intervals: from the start of monotherapy to the time of first disease progression/death, from the start of combination therapy to time of second disease progression/death, and from the start of monotherapy to the time of second disease progression/death; and safety.

### Assessments

Efficacy was evaluated according to RECIST v1.1; response was evaluated by the investigator. Tumor assessment by computed tomography (CT) scan was performed at baseline, every 6 weeks (two courses) after the start of treatment, and before the commencement of combination therapy in part B. Tumor assessment was not required prior to the start of combination therapy if a CT scan detecting progressive disease on afatinib monotherapy was conducted within 3 weeks prior to the commencement of combination treatment. However, if radiotherapy had been given prior to commencing combination therapy, a tumor assessment had to be performed to document the baseline status of combination therapy. Disease progression was determined based solely on clinical assessment or on both clinical judgment and tumor imaging. AEs and laboratory parameters were graded according to the Common Terminology Criteria for Adverse Events Version 3.0.

### Statistical analysis

Based on a binomial probability distribution and an assumed underlying objective response rate (ORR) of 10%, a total sample size of 80 patients would be expected to give an 82% probability of observing at least six responders. All analyses were descriptive and exploratory. PFS was assessed based on the Kaplan–Meier method for each part separately, and point estimates together with confidence intervals (CIs; based on Greenwood’s method) were calculated for median PFS.

## Results

### Patients

**Fig. 1:**
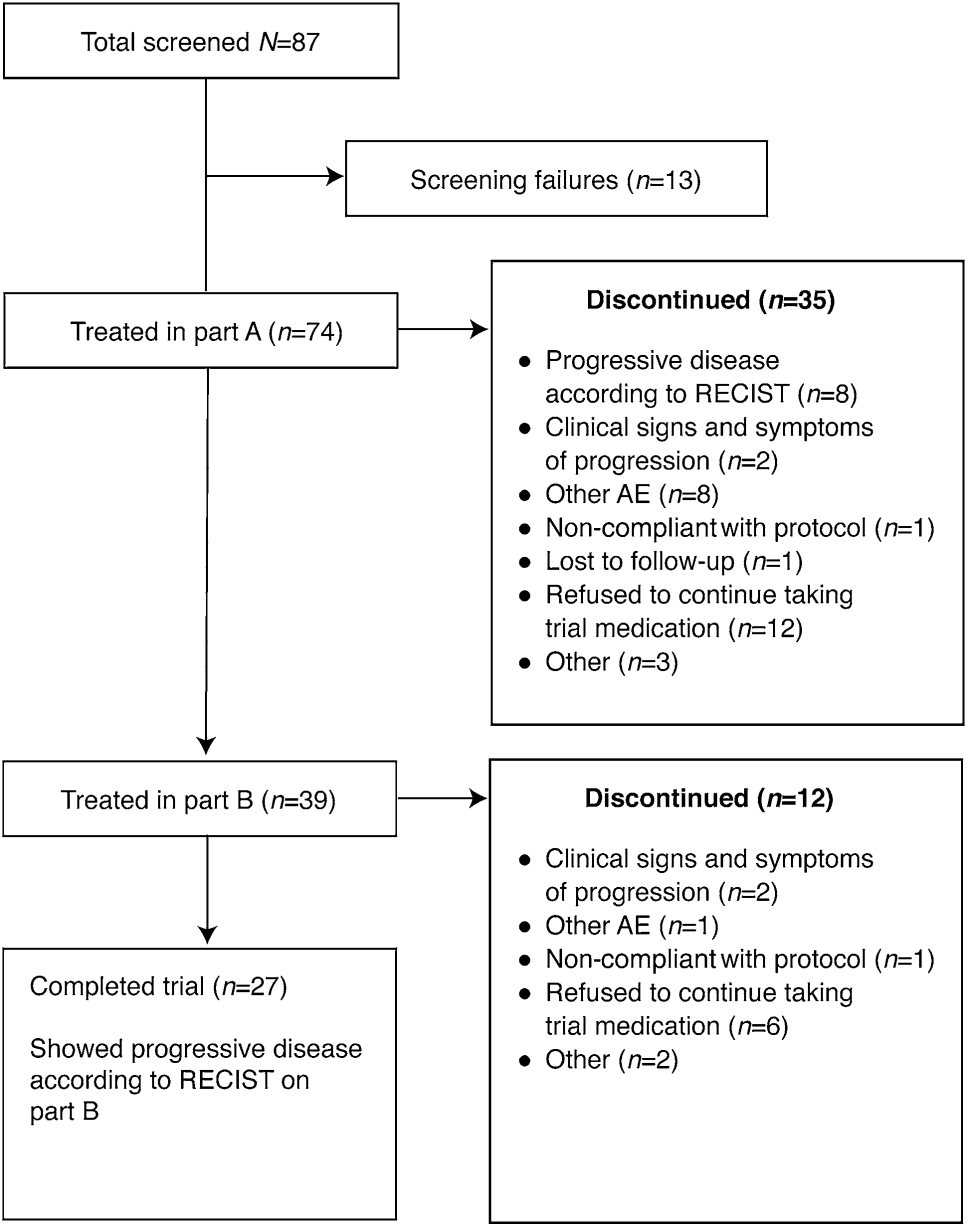
Patient disposition. *AE* adverse event, *RECIST* Response Evaluation Criteria in Solid Tumors

Between July 4, 2011 and October 16, 2013, 87 patients were enrolled, of whom 74 were treated in part A (Fig. 1). Of the 74 patients treated in part A, 39 (53%) went on to receive combination treatment in part B; 13 patients received afatinib and vinorelbine and 26 patients received afatinib and paclitaxel. While the study was ongoing, the data monitoring committee of a related trial, LUX-Breast 1, recommended termination of the afatinib and vinorelbine combination treatment arm in that study due to insufficient clinical benefit and higher rates of discontinuations, dose reductions, serious AEs, and deaths, relative to the comparator arm, trastuzumab, and vinorelbine [30]. As a precautionary measure, no further patients were enrolled into the afatinib and vinorelbine arm of this study from May 3, 2013. Later on, further enrollment into part A was also stopped. Patients who were receiving and benefitting from afatinib plus vinorelbine before May 3, 2013 were permitted to continue. Ongoing treatment in part A and treatment with afatinib plus paclitaxel in part B was not affected.

Baseline characteristics for patients in part A and part B are shown in Table 1. In total, 63 (85%) patients had infiltrating ductal carcinoma, six (8%) patients had infiltrating lobular carcinoma, one (1%) patient had tubular carcinoma, one (1%) patient had inflammatory breast cancer, and five (7%) patients had other tumor histology (multiple tumor types were reported for some patients).

**Table 1:** Baseline characteristics

Characteristic	Afatinib (part A; *N* = 74)	Afatinib + vinorelbine or paclitaxel (part B; *N* = 39)
Median age, years (range)	51 (27–76)	52 (27–73)
Race, *n* (%)		
Asian	47 (64)	22 (56)
Indian	16 (22)	5 (13)
Taiwanese or Chinese	31 (42)	17 (44)
White	27 (36)	17 (44)
Smoking status, *n* (%)		
Never smoked	69 (93)	37 (95)
Ex-smoker	2 (3)	1 (3)
Current smoker	3 (4)	1 (3)
Alcohol status, *n* (%)		
Non-drinker	63 (85)	32 (82)
Mean BMI, kg/m^2^ (SD)	25.8 (4.5)	27.1 (4.8)
ECOG PS, *n* (%)		
0	46 (62)	21 (54)
1	27 (36)	18 (46)
2	1 (1)	0
Menopausal status, *n* (%)		NE
Premenopausal	16 (22)	
Perimenopausal	3 (4)	
Postmenopausal	55 (74)	
Median time from first diagnosis, years (range)	2.4 (0.6–8.8)	NE
Estrogen receptor status at first diagnosis, *n* (%)		NE
Positive	31 (42)	
Negative	43 (58)	
Progesterone receptor status at first diagnosis^a^, *n* (%)		NE
Positive	26 (35)	
Negative	47 (64)	
HER2 status at first diagnosis, *n* (%)		NE
Positive	72 (97)	
Negative	2 (3)	
Previous HER2-targeted therapy		NE
Trastuzumab	64 (86)	
Lapatinib	6 (8)	
Trastuzumab and lapatinib	4 (5)	
Metastatic sites at baseline, *n* (%)		NE
1	23 (31)	
2	24 (32)	
3	17 (23)	
≥ 4	10 (14)	
Location of metastases, *n* (%)		NE
Lung	43 (58)	
Liver	32 (43)	
Skin	7 (9)	
Pleura	3 (4)	
Bone	14 (19)	
Lymph nodes	42 (57)	
Contralateral breast cancer	8 (11)	
Brain	1 (1)	
Other	8 (11)	

*BMI* body mass index, *ECOG PS* Eastern Cooperative Oncology Group performance status, *NE* not evaluated, *SD* standard deviation

^a^Data missing for one patient

Over the whole study (part A and part B combined), median exposure to afatinib was 166.0 days (range 1–1562 days). Median exposure to study medication was 83.5 days (range 1–1491 days) during part A, 92.0 days (range 29–266 days) in patients who received afatinib and vinorelbine in part B, and 128.5 days (range 1–1205 days) in patients who received afatinib and paclitaxel in part B.

### Efficacy

Thirteen (18%) patients had a confirmed objective response to afatinib monotherapy (part A), including one (1%) patient with a confirmed CR and 12 patients (16%) with a confirmed PR. Thirty-three (45%) patients had stable disease (Table 2). The patient with a confirmed CR was a 48-year-old Asian patient with moderately differentiated infiltrating ductal carcinoma. She had received prior treatment with lapatinib and tamoxifen and six courses of cyclophosphamide, doxorubicin, and 5-fluorouracil. CR was sustained for 211 days. Further two patients had an unconfirmed PR. Median duration of objective response (regardless of confirmation) was 168.5 days.

**Table 2:** Confirmed ORR

Patients, *n* (%)	Afatinib (part A; *N* = 74)	Afatinib + vinorelbine or paclitaxel (part B; *N* = 39)
Disease control (CR + PR + SD)	46 (62)	30 (77)
Objective response	13 (18)	12 (31)
CR	1 (1)	0
PR	12 (16)	12 (31)
SD	33 (45)	18 (46)
Unconfirmed CR/PR^a^	2 (3)	5 (13)
PD	21 (28)	4 (10)
Not evaluable	7 (9)	5 (13)

*CR* complete response, *ORR* objective response rate, *PD* progressive disease, *PR* partial response, *SD* stable disease

^a^CR/PR ≥ 35 days from first study drug administration in part A but not confirmed ≥ 28 days later

In part B, 12 (31%) patients had a confirmed PR during combined therapy with afatinib and vinorelbine or paclitaxel and a further five patients had an unconfirmed PR. Eighteen (46%) patients had stable disease. Median duration of objective response regardless of confirmation was 125.0 days.

**Fig. 2:**
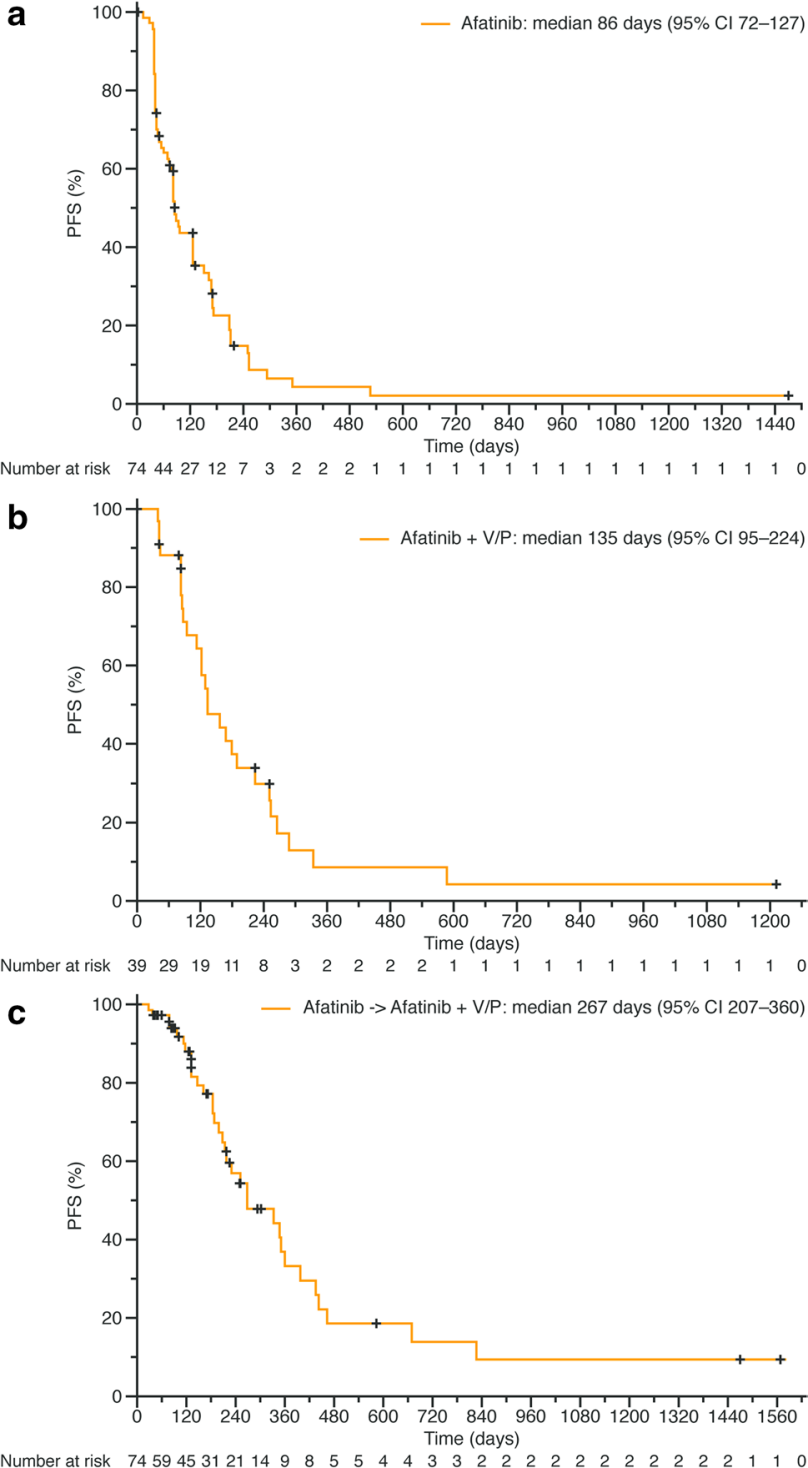
Progression-free survival during **a** part A (from the start of afatinib monotherapy to the time of the first disease progression or death); **b** part B (from the start of combined treatment with afatinib and vinorelbine or paclitaxel to the time of second disease progression or death); **c** the entire study (from the start of afatinib monotherapy to the time of second disease progression or death). *Afatinib* + *V/P* afatinib combination therapy with either vinorelbine or paclitaxel, *CI* confidence interval

Median PFS was 86 days (95% CI 72–127) in part A (Fig. 2a), 135 days (95% CI 95–224) in part B (Fig. 2b), and 267 days (95% CI 207–360) when considering the entire study (Fig. 2c).

### Safety

In total, 64 (86%) patients experienced treatment-related AEs in part A (Table 3). In part B, rates of treatment-related AEs were higher in patients receiving combination therapy with vinorelbine (92%) than with paclitaxel (85%). In total, 30 (41%) patients experienced an AE necessitating a reduction in the dose of afatinib during part A; fewer patients required dose reductions during part B (afatinib plus vinorelbine: 15%; afatinib plus paclitaxel: 27%; Table 3).

**Table 3:** Summary of adverse events in part A (afatinib) and part B (afatinib and vinorelbine or paclitaxel)

Patients, *n* (%)	Part A	Part B	
	Afatinib (*N* = 74)	Afatinib + vinorelbine (*N* = 13)	Afatinib + paclitaxel (*N* = 26)
Any AE	71 (96)	13 (100)	25 (96)
Treatment-related AE	64 (86)	12 (92)	22 (85)
AE leading to dose reduction	30 (41)^a^	2 (15)^b^	7 (27)^c^
AE leading to discontinuation	13 (18)^d^	2 (15)^e^	8 (31)^f^
Serious AE	18 (24)	5 (38)	10 (38)
Grade ≥ 3 AE	32 (43)	8 (62)	17 (65)

*AE* adverse event

^a^Most common: diarrhea (17 [23%]) and mucosal inflammation (3 [4%])

^b^Diarrhea and neutropenia

^c^Most common: diarrhea (4 [15%]) and neuropathy peripheral (2 [8%])

^d^Most common: diarrhea and neoplasm progression (2 [3%] each)

^e^Two patients experienced a total of three AEs: malignant neoplasm progression, neutropenia, and leukopenia

^f^Most common: asthenia, leukopenia, and vomiting (2 [8%] each)

The most common treatment-related AEs in patients receiving afatinib monotherapy in part A were (any/grade 3/4) diarrhea (68%/8%) and rash (49%/8%). In patients receiving afatinib with vinorelbine in part B, the most common treatment-related AE of any grade and of grade 3/4 was neutropenia (62%/38%). Among patients receiving afatinib with paclitaxel, the most common any grade treatment-related AEs were anemia (42%), alopecia (38%), and neutropenia (31%) and the most common grade 3/4 treatment-related AE was neutropenia (19%) (Table 4).

Four patients experienced treatment-related serious adverse events (SAEs) in part A: diarrhea (two patients), herpes zoster, and interstitial lung disease (one patient each). Among the patients receiving afatinib and vinorelbine in part B, two patients had a total of three treatment-related SAEs: abdominal pain, febrile neutropenia, and pyrexia. Two patients receiving afatinib and paclitaxel experienced a total of four treatment-related SAEs: azotemia, blood creatinine increased, diarrhea, and nausea.

Across parts A and B, 12 patients experienced fatal AEs, none of which were considered to be related to study treatment by the investigators. All fatal AEs represented disease progression and/or its complications.

**Table 4:** Most common treatment-related AEs

Number of patients, *n* (%)	Part A			Part B					
	Afatinib (*N* = 74)			Afatinib + vinorelbine (*N* = 13)			Afatinib + paclitaxel (*N* = 26)		
	Grade 1–2	Grade 3	Grade 4	Grade 1–2	Grade 3	Grade 4	Grade 1–2	Grade 3	Grade 4
Treatment-related AEs	44 (59)	20 (27)	0	6 (46)	4 (31)	2 (15)	12 (46)	9 (35)	1 (4)
Diarrhea	44 (59)	6 (8)	0	2 (15)	1 (8)	0	8 (31)	2 (8)	0
Neutropenia	1 (1)	0	0	3 (23)	3 (23)	2 (15)	3 (12)	5 (19)	0
Rash	33 (45)	3 (4)	0	2 (15)	0	0	3 (12)	0	0
Anemia	3 (4)	0	0	1 (8)	0	0	10 (38)	1 (4)	0
Alopecia	2 (3)	0	0	1 (8)	0	0	10 (38)	0	0
Fatigue	4 (5)	0	0	0	0	0	5 (19)	0	0
Asthenia	3 (4)	0	0	2 (15)	0	0	5 (19)	0	0
Leukopenia	0	0	0	3 (23)	0	0	5 (19)	0	0
Mucosal inflammation	13 (18)	1 (1)	0	0	0	0	1 (4)	0	0
Palmar-plantar erythrodysesthesia syndrome	10 (14)	2 (3)	0	1 (8)	0	0	2 (8)	1 (4)	0
Paronychia	9 (12)	0	0	2 (15)	0	0	1 (4)	0	0
Peripheral sensory neuropathy	0	0	0	0	0	0	4 (15)	0	0
Mouth ulceration	7 (9)	0	0	2 (15)	0	0	2 (8)	0	0
Nausea	5 (7)	0	0	0	0	0	3 (12)	1 (4)	0
Dermatitis	3 (4)	1 (1)	0	2 (15)	0	0	1 (4)	0	0
Neuropathy peripheral	0	0	0	0	0	0	3 (12)	0	0
Vomiting	3 (4)	0	0	0	0	0	2 (8)	0	1 (4)
AST increased	3 (4)	0	0	0	0	0	2 (8)	0	0
ALT increased	2 (3)	0	0	0	0	0	2 (8)	1 (4)	0
Erythema multiforme	0	2 (3)	0	0	0	0	0	0	0

AEs are presented by preferred term. No patient experienced a grade 5 treatment-related AE. Includes events reported for at least 10% of patients (grades 1–2) in any treatment group or any grade 3 or 4 event that was reported in more than one patient. Additional grade 3 AEs affecting one patient each in the afatinib monotherapy group were herpes zoster, interstitial lung disease, dermatitis acneiform, and skin ulcer. Additional grade 3 AEs affecting one patient each in the afatinib and paclitaxel group were skin fissures, skin ulcer, weight decreased, blood creatinine increased, and white blood cell count decreased. An additional grade 3 AE affecting one patient in the afatinib and vinorelbine group was pyrexia

*AEs* adverse events, *ALT* alanine aminotransferase, *AST* aspartate aminotransferase

## Discussion

When this study was conceived, options for patients with HER2-positive metastatic breast cancer who had failed treatment with taxanes and targeted agents were limited. We therefore investigated whether afatinib, given alone and in combination with vinorelbine or paclitaxel, could be a further treatment option for these patients. Despite the encouraging clinical activity seen in prior studies, enrollment was stopped after 74 patients had entered the study, following the discontinuation of a similar trial, LUX-Breast 1 [30]. This phase III study was terminated after a benefit–risk assessment indicated a low likelihood of improved PFS and lower tolerability, with afatinib and vinorelbine versus trastuzumab and vinorelbine. Similarly, in the phase II LUX-Breast 3 study, which enrolled patients with HER2-positive metastatic breast cancer with progressive brain metastases after trastuzumab, lapatinib, or both, no benefit and higher toxicity were seen with afatinib ± vinorelbine compared with investigators’ choice of treatment [31].

Despite the negative results seen in previous trials, findings from the present study suggest that afatinib monotherapy may be of clinical benefit to some patients with metastatic HER2-positive breast cancer who have failed prior HER2-targeted treatment. Overall, 62% of patients on afatinib monotherapy had a period of disease control, including one CR that was sustained for 211 days. Further, our data suggest that the combination of afatinib with vinorelbine or paclitaxel was also of clinical benefit to some patients who had progressed on afatinib monotherapy, with disease control achieved in 77% of patients.

The tolerability of afatinib and vinorelbine was generally comparable to that seen with afatinib and paclitaxel, with similar proportions of patients experiencing treatment-related grade ≥ 3 AEs in both treatment arms. Indeed, the incidence of treatment-related grade ≥ 3 AEs was only slightly higher in the afatinib combination arms than in the afatinib monotherapy group and the incidence of grade 1–2 events was lower AEs which were consistent with previous studies [25, 32–34] and no apparent adverse interactions between afatinib and vinorelbine or afatinib and paclitaxel were observed.

With phase III studies showing the clinical benefits of the newer anti-HER2 agents, pertuzumab, and trastuzumab emtansine [12, 13], the future role of afatinib for the treatment of HER2-positive patients who have previously been treated with trastuzumab appears to be limited. However, afatinib has shown some activity in other populations of patients with metastatic breast cancer, including patients with triple-negative breast cancer who had progressed following three or fewer lines of chemotherapy [35] and in combination with letrozole in patients with estrogen receptor-positive metastatic breast cancer who had progressed on letrozole monotherapy [36]. Further investigation into the use of afatinib in these settings may therefore be warranted; however, no further development of afatinib for HER2-positive breast cancer is currently planned. The findings from this study show that broad ErbB blockade may be of benefit in overcoming resistance to prior HER2-targeted therapy in only a small number of patients and suggest that, for most patients, novel agents targeting different oncogenic proteins and pathways are needed. Furthermore, now that we have entered the era of precision medicine and personalized medicine, there is still an ongoing urgent need for further specific markers for us to select the most optimal anti-HER2 therapy for this group of patients, as reflected by the achievement of a durable CR in a patient treated with single-agent afatinib in part A.

## Conclusion

Treatment with afatinib alone provided an objective response of 18% in patients with metastatic breast cancer who had previously failed or progressed on HER2-targeted therapy and objective response increased to 31% in patients whose disease progressed on afatinib monotherapy and were treated with afatinib in combination with vinorelbine or paclitaxel. These findings indicate that afatinib may provide some clinical benefit in this patient group. The AEs observed were in line with the known safety profile of afatinib. However, enrollment for this study was stopped early, following discontinuation of LUX-Breast 1 [30], for which a benefit–risk assessment favored trastuzumab in combination with vinorelbine over afatinib in combination with vinorelbine. No new trials for afatinib in HER2-positive breast cancer are planned; however, as afatinib has shown activity in other populations of patients with metastatic breast cancer [35, 36], further investigation in different specific indications together with companion biomarkers may be warranted.

## Data Availability

To ensure independent interpretation of clinical study results, Boehringer Ingelheim grants all external authors access to relevant material, including participant-level clinical study data, as needed by them to fulfill their role and obligations as authors under the ICMJE criteria. Clinical study documents and participant clinical study data are available to be shared on request after publication of the primary manuscript in a peer-reviewed journal, and if regulatory activities are complete and other criteria met as per the BI Policy on Transparency and Publication of Clinical Study Data (https://www.mystudywindow.com/msw/datasharing). Bona fide, qualified scientific and medical researchers are eligible to request access to the clinical study data with corresponding documentation describing the structure and content of the datasets. Upon approval, and governed by a Legal Agreement, data are shared in a secured data-access system for a limited period of 1 year, which may be extended upon request. Prior to providing access, clinical study documents and data will be examined, and, if necessary, redacted and de-identified, to protect the personal data of study participants and personnel, and to respect the boundaries of the informed consent of the study participants. Researchers should use the https://vivli.org/ link to request access to study data and visit https://www.mystudywindow.com/msw/datasharing for further information.
